# Distal tibial tubercle osteotomy can lessen change in patellar height post medial opening wedge high tibial osteotomy? A systematic review and meta-analysis

**DOI:** 10.1186/s13018-022-03231-0

**Published:** 2022-07-06

**Authors:** Yi-Ming Ren, Meng-Qiang Tian, Yuan-Hui Duan, Yun-Bo Sun, Tao Yang, Wei-Yu Hou

**Affiliations:** Department of Joint and Sport Medicine, Tianjin Union Medical Center, Nankai University Affiliated People’s Hospital, Jieyuan Road 190, Hongqiao District, Tianjin, 300121 People’s Republic of China

**Keywords:** Osteoarthritis, Tibial tubercle, Patella infera, High tibial osteotomy, Systematic review, Meta-analysis

## Abstract

**Objective:**

Medial opening wedge high tibial osteotomy (MOWHTO) is a mainstream surgical method for treating early medial compartment knee osteoarthritis. Undesirable sequelae such as patella infera may happen following tuberosity osteotomy. We conducted this systematic review and meta-analysis to compare the change in patellar position after proximal tibial tubercle osteotomy (PTO) versus distal tibial tubercle osteotomy (DTO) intervention.

**Methods:**

The 11 studies were acquired from PubMed, Medline, Embase and Cochrane Library. The data were extracted by two of the coauthors independently and were analyzed by RevMan5.3. Mean differences, odds ratios and 95% confidence intervals were calculated. Cochrane Collaboration’s Risk of Bias Tool and Newcastle–Ottawa Scale were used to assess risk of bias.

**Results:**

Eleven observational studies were assessed. The methodological quality of the trials ranged from moderate to high. The pooled results of postoperative patellar height (Caton-Deschamps index and Blackburne-Peel index) and postoperative complications showed that the differences were statistically significant between PTO and DTO interventions. Patellar index ratios decreased significantly in the PTO groups, and 12 (9.2%) complications under DTO surgery and 2 (1.6%) complications under PTO surgery were reported. The differences of postoperative posterior tibial slope (angle) was not statistically significant, but postoperative posterior tibial slope of both groups increased. Sensitivity analysis proved the stability of the pooled results and the publication bias was not apparent.

**Conclusions:**

DTO in MOWHTO maintained the postoperative patellar height, and clinically, for patients with serious patellofemoral osteoarthritis, DTO can be preferred. Postoperative complications are easily preventable with caution. In view of the heterogeneity and small sample size, whether these conclusions are applicable should be further determined in future studies.

## Introduction

Knee osteoarthritis (KOA) is a common degenerative disease, mainly characterized by slow progressive pain, swelling, stiffness and dysfunction of the knee joint [1]. A study that included individuals above 60 years of age in the USA has estimated the prevalence of radiographic changes consistent with KOA of the knee to be 37% [2]. At present, knee osteoarthritis cannot be completely cured. With the progression of the disease to the late stage, it has a great impact on the quality of life of patients. At this time, surgery is an effective treatment. Joint replacement is the main scheme for the treatment of severe KOA in the past. Although it can effectively reduce patients' pain and improve their joint function, it is not applicable to younger and more active patients who with minimal arthritis isolated to one medial compartment. In recent years, with the proposal of "knee preservation concept", osteotomy is more and more widely used in the treatment of KOA, mainly including medial opening wedge high tibial osteotomy (MOWHTO) and closed wedge high tibial osteotomy [3–5]. After a long time of development, MOWHTO is convenient and effective, and which has gradually become the mainstream surgical method for treating medial compartment KOA complicated with genu varus [6].

As for treatment of intraoperative tibial tubercle during MOWHTO, proximal tibial tubercle osteotomy (PTO) is a conventional surgical option [7–9]. However, gradually increasing patellofemoral osteoarthritis due to patella infera is reported as a common complication related to PTO [10–12]. In PTO, when the osteotomy surface is extended during operation, the tibial tubercle will migrate to the distal end, and the position of patella often decreases after operation. Then, patella tracking changes, which, in turn, lead to an increase in contact pressure and consequent cartilage degeneration in the PF joint. Lastly, this will affect the surgical effect of MOWHTO or remedial knee arthroplasty in future [13, 14]. The osteotomy surface of distal tibial tubercle osteotomy (DTO) is at the distal end of the tibial tubercle. Theoretically, the position of the tibial tubercle will not be changed when the osteotomy space is extended during the operation. So, it will not lead to the change of the position of the patella [15, 16]. Some studies have shown that DTO does not induce patella infera [17, 18]. Due to the different advantages and disadvantages of the above two osteotomies, there are some disputes about the choice of the two methods in clinic. This paper uses meta-analysis method to compare the postoperatively clinical safety of PTO and DTO in MOWHTO, in order to provide reference for the choice of clinical treatment strategy. According to our hypothesis, compared to biplanar ascending medial HTO, the descending HTO would preserve preoperative patellar height and posterior tibial slope parameters.

## Materials and methods

Ethical approval or patient consent was not required since the present study was a review of previous published literatures.

### Inclusive criteria of published studies

#### Types of studies

We considered all published and unpublished studies about DTO versus PTO with MOWHTO for treating knee osteoarthritis, covering randomized controlled trials (RCTs), and observational studies including retrospective and prospective studies.

#### Types of participants

The subjects were patients of KOA with clear diagnostic criteria and surgical indications. All patients had been diagnosed as patients of age greater than 18 years with radiographic evidence of isolated medial knee joint osteoarthritis who had failed conservative measures and were ready to accept osteotomy. There is no limit to the type of internal fixation or prosthesis used in the osteotomy. Exclusion criteria were multicompartmental arthritis; history of inflammatory arthritis; or history of prior surgery (aside from ligamentous repair) of the knee joint, distal femur, or proximal tibia.

#### Types of interventions

The operation method of the experimental group was DTO, and the operation method of the control group was PTO, which were considered. The exclusion criteria were as follows: (1) insufficient clinical outcome data in studies and (2) reviews, letters or conference articles.

#### Types of outcome measures

The primary outcome measures were the clinical outcomes synthesizing postoperative patellar height, and postoperative posterior tibial slope (angle). The secondary outcomes included: postoperative complications. Because the patellar height measured by Insal-Salvati method is the ratio of patellar length and patellar tendon length measured in sagittal position, while the patellar length and patellar tendon length are basically unchanged during osteotomy, which is not recommended to measure the patellar height of patients undergoing MOWHTO [19, 20]. The measurement methods of postoperative patellar height mainly include Caton-Deschamps method, Blackburne-Peel method. The normal values of Caton-Deschamps index (CDI); The normal value of Blackburne-Peel index (BPI) is 0.8 ~ 1.0, < 0.8 is the patella infera. Clinically, the patellar height is generally reduced by 10%, that is, the patellar height is significantly reduced.

### Search methods for identification of studies

Four databases (PubMed, Medline, Embase and Cochrane Library) were searched using the keywords such as “Osteoarthritis, Knee/knee osteoarthritis/KOA”, “proximal tibial tubercle osteotomy/PTO”, “distal tibial tubercle osteotomy/DTO”, “tibial tubercle”, “high tibial osteotomy/HTO” and “Randomized Controled Trial/RCT” through November 2021 to collect relevant studies about the clinical comparisons of DTO versus PTO with MOWHTO for treating KOA. The titles and abstracts of potential related articles identified by the electronic search were reviewed. References from retrieved articles were also assessed to extend the search strategy.

### Data collection and quality assessment

Two partners (YHD, YBS) independently assessed the titles and abstracts of all the studies screened during initial search, and they excluded any clearly irrelevant studies using the inclusion criteria. Data were independently extracted using a standard data form for the first author’s name, year of publication, sample size, gender, age, intervention, country, study design, follow-up and relevant outcome. A third partner (WYH) would handle any disagreement about inclusion of a study and reach a consensus. Cochrane Collaboration’s Risk of Bias Tool [21] was manipulated for the appraisal of RCT study quality. Observational studies were assessed by the Newcastle–Ottawa Scale including 8 items [22]. A higher overall score indicates a lower risk of bias and a score of 5 or less (out of 9) corresponds to a high risk of bias.

### Statistical analysis

RevMan statistical software v5.3 and Stata v16 were used for the meta-analysis. The analysis of continuous variables was conducted by mean difference (MD) and 95% confidence interval (CI). For a dichotomous outcome, we calculated the odds ratios (ORs) and 95% CIs. Heterogeneity was assessed by chi-squared and *I*^2^. A *P* < 0.05, *I*^2^ > 50% was considered significantly heterogeneous, and random-effect models were applied. Otherwise, fixed-effect models if there was no significant heterogeneity (*P* ≥ 0.05, *I*^2^ ≤ 50%). Sensitivity analysis was performed by omitting one study at a time to determine the stability of pooled results. Publication bias was determined by a funnel plot and statistically using the Egger test [23].

## Results

### Studies identification and inclusion

**Fig. 1:**
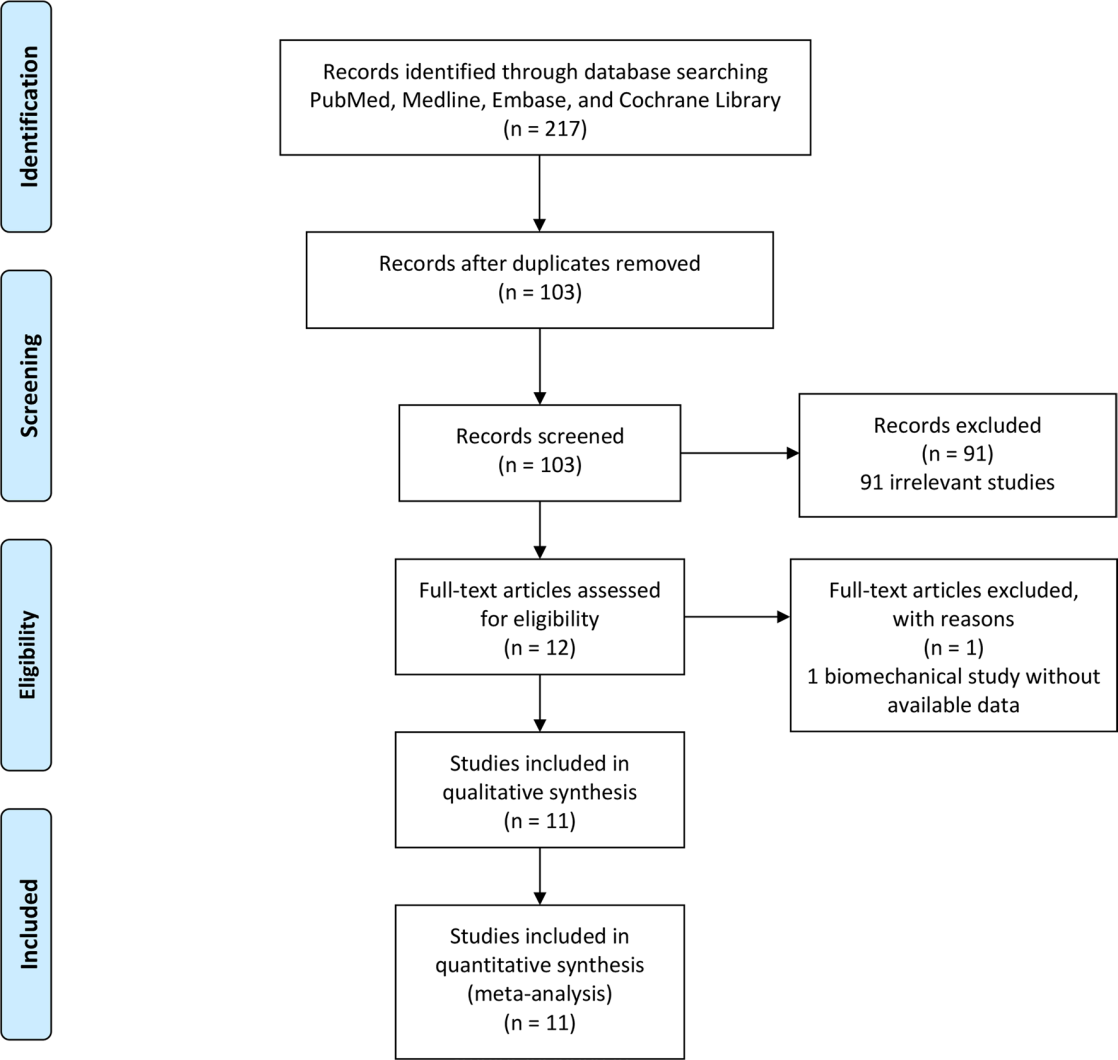
PRISMA flow diagram

Searches conducted in the PubMed, Medline, Embase and Cochrane Library, yielded a total of 217 articles. After removing duplicates, 103 literatures were remained. Based on the titles and abstracts review, 91 irrelevant articles were excluded. 12 full-text articles were assessed for eligibility. However, one article was excluded based on the previously established exclusion criteria (one biomechanical study without available data). Finally, 11 observational studies were included in this systematic review and meta-analysis. The detail of selection process is listed in Fig. 1.

### Study characteristics

We assessed 11 retrospective studies [24–34] in this article. The included studies were conducted in 7 countries (Japan, Germany, Korea, Singapore, Canada, Turkey and Netherlands) from 2004 to 2019, and involved 7339 patients (332 patients treated with DTO technique, 407 patients treated with PTO technique) aged 30.5 to 63.0 years. The average follow-up duration ranged from 1.5 to 48 months. The clinical outcomes of the studies were evaluated mainly based on the postoperative patellar height, postoperative posterior tibial slope (angle), and postoperative complications. The detailed information of included studies is shown in Table 1.

**Table 1:** Characteristics of studies included

	Year	Sample size (DTO/PTO)	Female (%)	Mean age(years)	Intervention DTO PTO		Country	Study design	follow-up (month)	Relevant outcome
Kim et al .[24]	2021	44/50	66.0	DTO 56.83 ± 5.93 PTO 55.88 ± 7.02	Distal tibial tuberosity osteotomy	Proximal tibial tubercle osteotomy	Korea	Retrospective study	48	Postoperative patellar height; posterior tibial slope; postoperative complications
Horikawa et al .[25]	2019	46/65	67.6	DTO 62.6 ± 6.2 PTO 63.0 ± 7.1	Distal tibial tuberosity osteotomy	Proximal tibial tubercle osteotomy	Japan	Retrospective study	12	Postoperative patellar height;FTA; JOA score;posterior tibial slope
Ogawa et al.[26]	2019	43/41	66.7	DTO 62.3(40–73) PTO 62.8(48–75)	Distal tibial tuberosity osteotomy	Proximal tibial tubercle osteotomy	Japan	Retrospective study	DTO 22.2 PTO 33.7	Postoperative patellar height; KSS
Krause et al. [27]	2017	32/32	37.5	45.2 ± 8.7	Distal tibial tuberosity osteotomy	Proximal tibial tubercle osteotomy	Germany	Retrospective study	NR	Postoperative patellar height;FTA; posterior tibial slope
Park et al .[28]	2017	33/30	66.7	DTO 30.5 ± 8.0 PTO 32.8 ± 7.5	Distal tibial tuberosity osteotomy	Proximal tibial tubercle osteotomy	Korea	Retrospective study	DTO 33.1 ± 2.9 PTO 32.4 ± 8.7	Postoperative patellar height;FTA; posterior tibial slope; postoperative complications
Gooi et al .[29]	2017	24/82	37.7	48.8 ± 10.8	Distal tibial tuberosity osteotomy	Proximal tibial tubercle osteotomy	Singapore	Retrospective study	NR	Postoperative patellar height; posterior tibial slope
Morsi et al .[30]	2014	25/25	50	DTO 48(41–59) PTO 47.7(42–58)	Distal tibial tuberosity osteotomy	Proximal tibial tubercle osteotomy	Egypt	Retrospective study	DTO 29.1(12–36) PTO 27.4(12–34)	Postoperative patellar height;KSS postoperative complications
Longino et al. [31]	2013	29/29	27.6	DTO 46 ± 8 PTO 49 ± 6	Distal tibial tuberosity osteotomy	Proximal tibial tubercle osteotomy	Canada	Retrospectivestudy	DTO 23.1 ± 6.6PTO 22.7 ± 6.1	Postoperative patellar height;posterior tibial slope
Elmali et al .[32]	2012	26/21	78.7	DTO 55 ± 7PTO 55 ± 9	Distal tibial tuberosity osteotomy	Proximal tibial tubercle osteotomy	Turkey	Retrospectivestudy	DTO 38 ± 5PTO 40.6 ± 7	HSS;FTA;postoperative patellar height;posterior tibial slope;postoperativecomplications
Hinterwimmer et al .[33]	2011	13/12	12	40.2 ± 8.9	Distal tibial tuberosity osteotomy	Proximal tibial tubercle osteotomy	Germany	Retrospectivestudy	NR	Postoperative patellar height;posterior tibial slope
Gaasbeek et al .[34]	2004	17/20	24.3	DTO 48 ± 10PTO 42 ± 11	Distal tibial tuberosity osteotomy	Proximal tibial tubercle osteotomy	Netherlands	Retrospectivestudy	1.5	Postoperative patellar height;

*PFO *proximal tibial tubercle osteotomy; *DTO *distal tibial tuberosity osteotomy; *JOA *Japanese orthopaedic association; *KSS *knee society score; *HSS *hospital for special surgery knee score, index, *FTA *femur-tibia angle; *NR *not reported

### Methodological assessment of study quality

Methodological quality assessment of the 11 included studies is presented in Table 2. Among the observational studies, the Newcastle–Ottawa Scale including the exposed cohort, the non-exposed cohort, ascertainment of exposure, outcome of interest, comparability, assessment of outcome, length of follow-up and adequacy of follow-up, was used to assess the risk of bias. The scores of all 11 studies [24–34] were all 6 to 8, indicating a low risk of bias.

**Table 2:** Newcastle–Ottawa Scale of observational studies

		Selection					Outcome		
Study	Exposed Cohort	Noexposed Cohort	Ascertainment of Exposure	Outcome of Interest	Comparability	Assessment of Outcome	Length of Follow-Up	Adequacy of Follow-Up	Total Score
Kim et al .[24]	*	*	*	*	*	*	*	*	8
Horikawa et al .[25]	*	*	*	*	*	*	*	*	8
Ogawa et al .[26]	*	*	*	*	*	*	*	*	8
Krau se et al .[27]	*	*	*	*	*	*			6
Park et al .[28]	*	*	*	*	*	*	*	*	8
Gooi et al .[29]	*	*	*	*	*	*			6
Morsi et al .[30]	*	*	*	*	*	*	*	*	8
Longino et al .[31]	*	*	*	*	*	*	*	*	8
Elmali et al .[32]	*	*	*	*	*	*	*	*	8
Hinterwimmer et al .[33]	*	*	*	*	*	*			6
Gaasbeek et al. [34]	*	*	*	*	*	*	*		7

* Risk of bias was assessed using the Newcastle–Ottawa Scale. A higher overall score indicates a lower risk of bias; a score of 5 or less (out of 9) corresponds to a high risk of bias

### Comparison of Caton-Deschamps index (CDI) between DTO and PTO

**Fig. 2:**
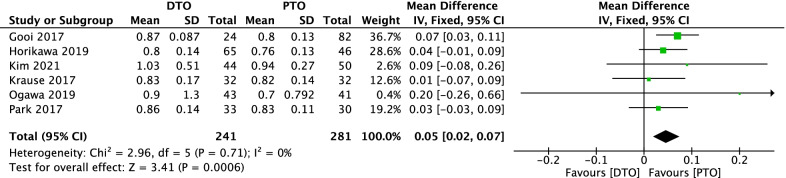
Forest plot of comparison: Caton-Deschamps index (CDI) between proximal tibial tubercle osteotomy (PTO) versus distal tibial tubercle osteotomy (DTO) for knee osteoarthritis (KOA)

Comparison of CDI between DTO and PTO was conducted among the 6 included studies [24–29], which included 522 patients (241 patients receiving DTO and 281 patients receiving PTO), as shown in Fig. 2. Heterogeneity testing showed that there was none heterogeneity among the studies (*P* = 0.71, *I*^2^ = 0%), so the fixed-effect model was used to pool the data from the 6 studies. The pooled result showed that the difference was statistically significant between the DTO group and the PTO group (MD = 0.05, 95% CI = 0.02–0.07, *P* = 0.0006).

### Comparison of Blackburne-Peel index (BPI) between DTO and PTO

**Fig. 3:**
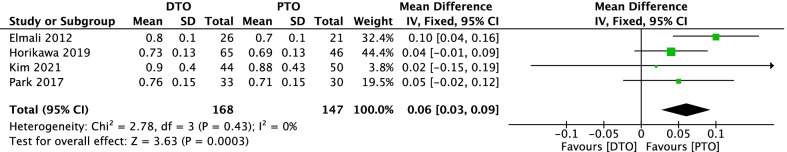
Forest plot of comparison: Blackburne-Peel index (BPI) proximal tibial tubercle osteotomy (PTO) versus distal tibial tubercle osteotomy (DTO) for knee osteoarthritis (KOA)

In Fig. 3, four included studies [24, 25, 28, 32] consisting of 315 patients (168 patients received DTO treatment and 147 patients received PTO treatment) investigated BPI. No heterogeneity among studies (*P* = 0.43, *I*^2^ = 0%) was found, so we used the fixed-effect model to pool the data. The overall estimate showed that the difference was statistically significant between the PTO group and the DTO group (MD = 0.06, 95% CI = 0.03–0.09, *P* = 0.0003).

### Comparison of postoperative posterior tibial slope between DTO and PTO

**Fig. 4:**
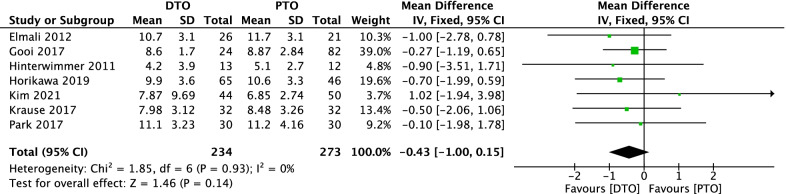
Forest plot of comparison: postoperative posterior tibial slope between proximal tibial tubercle osteotomy (PTO) versus distal tibial tubercle osteotomy (DTO) for knee osteoarthritis (KOA)

Comparison of postoperative posterior tibial slope between DTO and PTO treatment was conducted among 7 included studies [24, 25, 27–29, 32, 33] which contain 507 patients in Fig. 4. A heterogeneity test showed that there was none heterogeneity among studies (*P* = 0.93, *I*^2^ = 0%), so the fixed-effect model was used. The overall estimate showed that the difference between the two groups was not statistically significant (MD = − 0.43, 95%CI = − 1.00–0.15, *P* = 0.14).

### Comparison of postoperative complications DTO and PTO

**Fig. 5:**
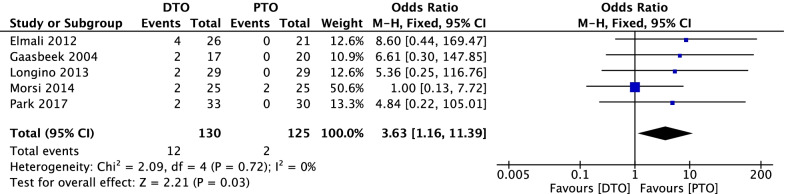
Forest plot of comparison: postoperative complications between proximal tibial tubercle osteotomy (PTO) versus distal tibial tubercle osteotomy (DTO) for knee osteoarthritis (KOA)

In Fig. 5, 5 included studies [28, 30–32, 34] consisting of 255 OA patients (130 patients received DTO and 125 patients received PTO technique) reported postoperative complications. On the whole, 12 (9.2%) complications under DTO surgery were reported and 2 (1.6%) complications under PTO surgery were reported in 5 included studies [28, 30–32, 34]. The major complications reported after DTO surgery included fracture of the lateral tibial plateau, tibial tuberosity fracture, delayed healing, superficial infection, and lower extremity deep venous thrombosis. No heterogeneity among studies (*P* = 0.72, *I*^2^ = 0%) was found, so we used the fixed-effect model. The overall estimate indicated that the pooled OR was 3.63 (95%CI = 1.16–11.39, *P* = 0.03), suggesting that the difference was statistically significant between DTO intervention and PTO intervention.

### Sensitivity analysis and publication bias

**Fig. 6:**
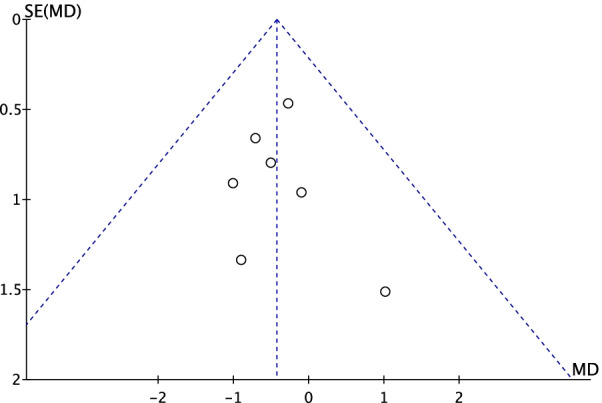
Funnel plot to test for publication bias. Each point represents a separate study for the indicated association. The vertical line represents the mean effects size. MD = mean difference; SE = standard error

We performed a sensitivity analysis to assess the stability of the pooled results. Among the most studies, the heterogeneity results were not obviously altered after sequentially omitting each study, indicating that our results were statistically reliable. The funnel plot of the included studies is shown in Fig. 6. The points in the funnel plot were almost symmetrically distributed, and Egger test *P* = 0.817 indicating that the publication bias was not apparent.

## Discussion

### Summary of main results

Postoperative patella infera is a common complication of MOWHTO with PTO [10–12]. The osteotomy slope of the descending MOWHTO is located on the patellar tubercle. When the osteotomy surface is extended during the operation, the tibial tubercle will migrate to the distal end, and the tibial tubercle is the attachment point of the patellar tendon. Therefore, the position of the patella often decreases after the operation [33, 35]; In addition, the greater the degree of intraoperative correction of varus deformity, the higher the possibility of patella infera after operation. The lower the position of patella, the greater the pressure of patellofemoral joint in the process of knee flexion, which is easy to accelerate the degeneration of patellofemoral joint [36]. In view of this, in recent years, the MOWHTO infra the tibial tubercle has gradually been used in the treatment of genu varus with medial compartment KOA. Theoretically, the osteotomy slope of this operation is located under the tibial tubercle. Some studies had proved that when the osteotomy space is extended during the operation, the position of the tibial tubercle will not be changed, so it will not lead to the change of the position of the patella [17, 18, 27, 28]. Through meta-analysis, this study compared the patellar height measured by Caton-Deschamps method and Blackburne-Peel method. Pooled-results showed that patellar index ratios decreased significantly in the PTO groups, and the MOWHTO above the tibial tubercle was more likely to lead to patella infera after operation.

The meta-analysis results also revealed that there is no statistically significant difference between PTO and DTO, and both PTO and DTO with MOWHTO will lead to the increase in posterior tibial slope after operation. On the current research view, the increase in postoperative posterior tibial slope will increase the tension of anterior cruciate ligament (ACL), so as to accelerate the degeneration or injury of ACL. By arthroscopic view of the degenerative changes in the ACLs, Kim et al. and Ogawa et al. confirmed increased posterior tibial slope following MOWHT resulted in degenerative ACL changes. Greater body mass index and larger difference in posterior tibial slope (angle) were predisposing factors for ACL degeneration after MOWHTO [37, 38]. Ogawa et al. also found that the magnitude of change in the medial proximal tibial angle is correlated with ACL degeneration following MOWHTO, causing that the reduction of coronal tibiofemoral subluxation following MOWHTO lengthens ACL in the coronal plane and accelerated ACL degeneration [39]. What’s more, geometric changes of the joint line orientation angle, medial femoral notch to medial spine length on the coronal plane, and posterior tibial slope on the sagittal plane after OWHTO were related to ACL deterioration. The ACL was commonly affected at the middle and distal portions and rarely at the proximal portion. There is a possibility of impingement because of the geometric changes [40]. There is no unified understanding of why MOWHTO is easy to lead to the increase in postoperative posterior tibial slope. NHA et al. [41] believe that the increase in postoperative posterior tibial slope is related to the position of osteotomy spreader during MOWHTO. If the spreader is placed forward, posterior tibial slope is easy to increase after operation. Some researchers believe that after operation the increase in posterior tibial slope is related to the geometry of tibia and the position of lateral hinge [42–44]. Yong et al. [43] studied the relationship between the integrity of the lateral hinge and posterior tibial slope after MOWHTO by using three-dimensional CT. The results showed that in patients with posterolateral hinge fracture, the postoperative posterior tibial slope increased significantly, while in patients with complete hinge or fracture location, there was no significant difference in the change of posterior tibial slope before and after operation. Jo et al. [44] found that the position of the lateral hinge affects the change of posterior tibial slope. The lower the position of the lateral hinge, the more obvious the increase in postoperative posterior tibial slope. Ogawa et al. [26] also found that the increase in postoperative posterior tibial slope is related to the position and direction of the lateral hinge. In order to avoid the increase in posterior tibial slope after operation, the posterior osteotomy space can be maintained to be twice the anterior osteotomy space during operation, or the insertion direction of chisels can be changed and fine adjustment can be made after intraoperative fluoroscopy measurement of posterior tibial slope [37, 45].

Although the MOWHTO above the tibial tubercle is easy to lead to the postoperative patella infera, the results of Elmali’s study [32] show that post-operative hospital for special surgery knee score (HSS) scores increased significantly and there is no significant difference in HSS between the DTO group and the PTO group, indicating that the two have similar early clinically efficacy in the treatment of medial compartment KOA.

The complications in nine included studies also should be discussed. 12 (9.2%) complications under DTO surgery were reported and 2 (1.6%) complications under PTO surgery were reported in 5 included studies [28, 30–32, 34], which showed that PTO treatment has the lower complication rate than DTO treatment. Some studies reported that the postoperative complication rate of MOWHTO was 0 ~ 37%, and the major complications after MOWHTO surgery included superficial peroneal nerve traction injury, neurovascular injury, patella baja, infection, numbness, knee instability, delayed healing, lower extremity deep venous thrombosis, pulmonary embolism, hinge fracture, recurrence deformity and so on [6, 46]. Park et al. hold that tibial tuberosity fracture is related to mistake of surgical procedure and easily preventable with caution [28]. Tibial tubercle fracture did not affect the clinical outcome and bony union in spite of the relatively high occurrence rate. Anatomical risk factors for the fractures are a lower tibial tubercle position [47]. A biomechanical study demonstrated that distal tibial tuberosity arc osteotomy (DTAO) increased the flange contact area and decreased the wedge volume in the opening gap compared to DTO, which would be an advantage for bone union at the osteotomy site [48].

### Limitations of the study

Some limitations of this study should be noted. First, the small sample size might have affected the significant difference between the two surgical procedures. Second, it was based merely on clinical findings and measurements at 1.5–48 mouths postoperative which may underestimate the reported number of complications. Thus, a longer follow-up study with final functional outcomes evaluation will be more meaningful. Third, the included studies were observational studies and not RCTs, and they largely relied on retrospectively collected data, resulting in a high risk of selection bias. More large-sample, multi-center, high-quality, randomized controlled trials are needed to verify the outcomes of this meta-analysis.

## Conclusions

In conclusion, the results of this study suggest that during MOWHTO, both PTO and DTO increase posterior tibial slope after operation; The former is more likely to cause postoperative patella infera than the latter, but its safety is higher than the latter. Clinically, for patients with serious patellofemoral osteoarthritis, DTO can prevent the complication of patella baja and be preferred. In view of the heterogeneity and small sample size, whether these conclusions are applicable should be further determined in future studies.

## Data Availability

The present study was a review of previous published literatures.

## Abbreviations

KOA: Knee osteoarthritis
MOWHTO: Medial opening wedge high tibial osteotomy
PTO: Proximal tibial tubercle osteotomy
DTO: Distal tibial tubercle osteotomy
RCT: Randomized controlled trial
CDI: Caton-Deschamps index
ISI: Insal-Salvati index
BPI: Blackburne-Peel index
MD: Mean difference
CI: Confidence interval
OR: Odds ratio
ACL: Anterior cruciate ligament
HSS: Hospital for special surgery knee score
DTAO: Distal tibial tuberosity arc osteotomy
